# Microglia Are Essential to Protective Antiviral Immunity: Lessons From Mouse Models of Viral Encephalitis

**DOI:** 10.3389/fimmu.2019.02656

**Published:** 2019-11-13

**Authors:** Catherine F. Hatton, Christopher J. A. Duncan

**Affiliations:** ^1^Immunity and Inflammation Theme, Institute of Cellular Medicine, Newcastle University, Newcastle upon Tyne, United Kingdom; ^2^Department of Infection and Tropical Medicine, Newcastle upon Tyne Hospitals NHS Foundation Trust, Newcastle upon Tyne, United Kingdom

**Keywords:** viral immunity, microglial depletion, CSF1R, PLX5622, IL34, interferon, neuroimmunology, central nervous system

## Abstract

Viral encephalitis is a rare but clinically serious consequence of viral invasion of the brain and insight into its pathogenesis is urgently needed. Important research questions concern the involvement of the host innate immune response in pathogenesis, key to which is the role played by microglia, resident macrophages of the brain parenchyma. Do microglia have a protective function, by coordinating the innate immune response to viral infection, or do they drive pathogenic neuroinflammation? Here we synthesize recent data from mouse models of acute viral encephalitis, which reveal an unambiguously protective role for microglia. Depletion of microglia, via blockade of colony-stimulating factor 1 receptor (CSF1R) signaling, led to increased viral replication accompanied by more severe neurological disease and heightened mortality. Whilst the underlying mechanism(s) remain to be defined, microglial interactions with T cells and phagocytosis of infected neurones appear to play a role. Paradoxically, the production of inflammatory cytokines was increased in several instances following viral infection in microglia-depleted brains, suggesting that: (i) cells other than microglia mediate inflammatory responses and/or (ii) microglia may exert a regulatory function. Under certain circumstances the microglial antiviral response might contribute negatively to longer-term neurological sequelae, although fewer studies have focused on this aspect in encephalitis models. Understanding regulation of the microglial response, and how it contributes to disease is therefore a priority for future studies. Collectively, these findings demonstrate the central role of microglia in pathogenesis, suggesting the exciting possibility that defects of microglial function might contribute to encephalitis susceptibility and/or outcome in humans.

## Introduction

Viral encephalitis is defined as pathological inflammation of the brain parenchyma secondary to viral infection (1). This syndrome is a rare but clinically serious outcome of infection with a range of DNA and RNA viruses. Encephalitis is associated with appreciable mortality and high rates of permanent neurological impairment in survivors, and in most cases there is no available antiviral therapy (1). Annual healthcare expenditure associated with the acute care of patients with encephalitis was estimated in the region of $2 billion (2), although indirect costs are likely much higher. Furthermore, several emerging causes of viral encephalitis are considered by WHO to be a significant threat to global public health (3). Fundamental to addressing this unmet medical need is research to better understand the pathogenesis of viral encephalitis (4).

## Host Innate Immunity: Protective or Pathogenic in Viral Encephalitis?

Whilst some viruses are highly neurovirulent (e.g., rabies virus), most people infected with neurotropic viruses do not go on to develop encephalitis, suggesting that rare host and/or viral factors underlie susceptibility. Defining these factors is a major challenge for the field. Despite decades of investigation, there is limited evidence of viral determinants of neurovirulence (1, 5), implicating the host response in pathogenesis. However, it remains unclear whether immunity plays a predominantly beneficial or deleterious role. In other words, do patients develop encephalitis because their response to viral infection is inadequate, impairing antiviral resistance, or excessive, resulting in immunopathology? There is evidence from human studies to support both hypotheses. Although not necessarily mutually exclusive, they imply opposite therapeutic approaches (i.e., cytokine supplementation vs. immunosuppression).

The hypothesis that encephalitis arises through a failure of host resistance is supported by very rare genetic errors of innate antiviral immunity, which confer heightened susceptibility to encephalitis secondary to wild-type (6–14) or attenuated vaccine strain viruses (15, 16). These disorders provide compelling evidence that under normal circumstances innate immunity provides a critical layer of protection against viral encephalitis. The extent to which inborn errors of innate immunity underlie encephalitis more generally, including in adults, remains to be determined.

The alternative hypothesis, that pathogenesis is governed not by a failure to resist viral replication, but rather due to excessive innate immune-mediated damage, is supported by: (i) a lack of correlation between viral loads in cerebrospinal fluid and disease outcome (17–20), suggesting that the extent of viral replication does not alone dictate pathology; and (ii) correlations between biomarkers of innate immune activation and poor outcome (21–23). In addition, phenotypic overlaps are recognized between congenital viral diseases such as cytomegalovirus (CMV) and the genetic disease Aicardi-Goutières syndrome (24), in which the aberrant induction of antiviral innate immunity is considered central to pathogenesis (25); the implication of this shared phenotype is that much of the neurological damage arising from congenital viral infection may be host-derived (26). Nevertheless, there remains no conclusive proof of a causal link between immune activation and neurological outcome, since it is impossible in clinical studies to separate the effects of viral cytopathicity and immunopathology. Insight into this question may come from an ongoing clinical trial of anti-inflammatory corticosteroids in patients with herpes simplex virus encephalitis (https://clinicaltrials.gov/ct2/show/NCT03084783) (27).

Mouse models of viral encephalitis provide an alternative means to resolve these issues. Whilst there are caveats to the use of mice to understand human disease (28, 29), key pathological features of encephalitis are reproduced in these models (30, 31), which have the advantage of being amenable to controlled experimental perturbation. A key theme to emerge from recent studies is the critical role of antiviral immunity within the brain itself.

## A Brain Intrinsic Antiviral Interferon Network Combats Viral Spread in the Brain

Contrary to the long-held view of the brain as an immune-privileged organ, it is now clear that the brain parenchyma poses an intrinsic antiviral network, in which the antiviral cytokines known as type I interferons (IFNs) play a central role (32, 33). Detje et al. were the first to show that the response of neuroectodermal cells (including neurones, astrocytes, and oligodendrocytes) to type I IFNs was essential for the protection of mice against vesicular stomatitis virus (VSV) encephalitis (34). These findings were subsequently reproduced in a model of herpes simplex virus (HSV1) encephalitis (35). All cell types of the central nervous system (CNS) are capable of mounting a type I IFN response, although the relative efficiency of this process appears to vary substantially (33, 36). In VSV models, interferon alpha/beta receptor (IFNAR) signaling within olfactory neurones (37) and astrocytes (38) was necessary to limit viral dissemination throughout the CNS, suggesting that type I IFNs act on viral target cells to control permissiveness and/or onward transmission. However, the cell type(s) responsible for initiating the production of type I IFNs, and the precise intercellular signaling events that underpin protection, remain to be determined. Astrocytes were reported to be the main producers of IFNβ upon infection with model neurotropic RNA viruses (39, 40). Simultaneously, data generated in other encephalitis models have rekindled interest in the role of microglia (32, 41, 42).

## Microglia: at the Hub of the Brain's Antiviral Network?

It has long been suspected that microglia—the sole brain-resident immune cells—play an essential role in antiviral defense of the brain (43). However, the tools were not previously available to formally test this hypothesis. Microglia are the “third element” of the CNS, initially described by Ramón y Cajal and further characterized by Del Río-Hortega. These cells are parenchymal resident macrophages of the brain parenchyma, arising from embryonic yolk sac precursors (44, 45) which seed the developing brain around embryonic day 9.5. This period coincides with neuronal birth and is before the formation of the blood-brain barrier and the development of astrocytes or oligodendrocytes (46). Thus, microglia fundamentally shape the developing brain by supporting neurogenesis (47, 48) and synaptic remodeling (49, 50). The essential role of microglia in normal brain development is revealed by humans (51, 52) and mice (44, 53) with genetic deficiencies of microglia due to homozygous deficiency of the colony-stimulating factor 1 receptor (CSF1R).

In the current paradigm, microglia self-renew under homeostatic conditions with minimal contribution from circulating monocytes (44, 54). Like other tissue-resident macrophages, microglia respond rapidly to environmental cues with a broad spectrum of activation states (55). They express relevant endosomal and cytosolic pathogen pattern recognition receptors for detection of viral molecules and are capable of efficiently sensing viral pathogens *in vitro* (56). Reactive microgliosis, defined an increase in microglial numbers and a change from ramified to amoeboid morphology, is observed in both patients and mice with viral encephalitis (56, 57), and appears to be dependent to some extent on IFNAR signaling (42, 58). Whether microglial reactivity is an appropriate response to viral invasion, or contributes negatively to disease, continues to provoke debate (56, 59). Prior to the availability of efficient methods of microglial depletion, studies reported both protective (41, 60) and pathogenic (61, 62) effects of microglia in encephalitis models. Since then, considerable progress has been made in resolving this controversy through the use of targeted depletion of microglia in mouse models of encephalitis. Methods of microglial depletion have been comprehensively reviewed elsewhere (63, 64) and are briefly summarized below.

## Methods of Microglial Depletion

The original approach, developed in the 1980s, was an infusion of clodronate-encapsulated liposomes, which are toxic to macrophages (65). Since liposomes are not capable of penetrating the blood-brain barrier, intracranial or intraventricular administration is necessary to deplete microglia (66, 67). However, even under these circumstances, depletion is incomplete. Various genetic methods were subsequently developed that permitted more efficient targeting of microglia (63), however these have not been used in encephalitis models. By contrast, blockade of CSF1R signaling has been more widely adopted.

**Figure 1:**
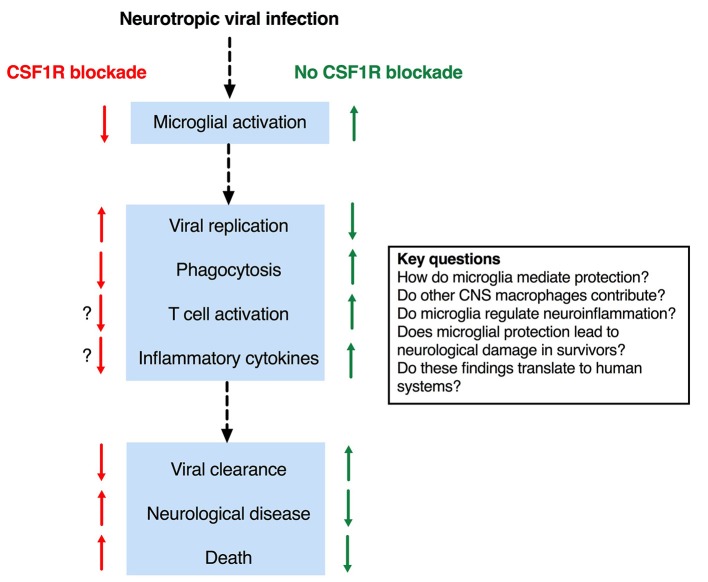
Effects of microglial depletion in mouse models of viral encephalitis and key outstanding questions.

As alluded to above, microglia are dependent on signaling through the CSF1R for development and survival (33, 44, 51, 53). *Csf1r*^−/−^ mice lack microglia as well as all tissue macrophage populations (44, 53). However, these mice also exhibit profound developmental defects and significant perinatal mortality, making them unsuitable for studies of encephalitis. The ligands for CSF1R are CSF1 and IL34. Deletion of either does not lead to complete loss of microglia in the brain, reflecting a degree of redundancy (63). *Il34*^*LacZ*/*LacZ*^ reporter mice (which are effectively IL34 deficient) have been used in a limited number of studies of viral encephalitis (68, 69). Blockade of CSF1R [e.g., with the small molecules such as PLX5622 (70, 71)] efficiently depletes microglia from the brain of intact rodents (72) and has been more frequently employed, presumably due to its technical ease. Collectively, the results of these studies provide a coherent set of data that supports a protective role for microglia in the acute phase of viral encephalitis (Figure 1).

## Microglia Are Protective in Encephalitis Disease Models

A range of neurotropic viruses from several families have been studied in microglial depletion models (Table 1). Despite the diversity of viruses investigated, consistent findings have emerged. In virtually all cases, the replication of neurotropic viruses was enhanced by depletion of microglia (58, 73–79), indicating that microglia are essential for viral resistance in the CNS. The only exception was *Il34*^*LacZ*/*LacZ*^ mice, in which viral replication was unaffected (68). In this model, microglia numbers were only modestly reduced in the cerebral cortex (3-fold), and unchanged in the cerebellum (68).

**Table 1:** Microglial depletion studies in mouse models of viral encephalitis.

**Study**	**Strain**	**Model**	**Sex**	**Prior to infection**	**Virus family**	**Virus (route)**	**Mort**.	**Morb**.	**Viral burden**	**Cytokines**	**Protective mechanism(s)**
Wang	C57/B6	*Il34^*LacZ*/*LacZ*^*	M/F	n/a	*Flaviviridae*	WNV (i.c.) WNV-E218A (i.c.)	↑	↑	↔	n/r	Immunoregulation
Tsai	ICR (neonates)	CLD	n/r	2 d	*Flaviviridae*	DENV (i.c. and i.p.)	↑	↑	↑	↓	Cytokine production; CTL recruitment/activation
Vasek	C57/B6	*Il34^*LacZ*/*LacZ*^*	M/F	n/a	*Flaviviridae*	WNV-E218A (i.c.)	n/r	n/r	n/r	n/r	n/a C3 mediated phagocytosis
Wheeler	C57/B6	PLX5622	M	7 d	*Coronaviridae*	MHV (i.n.)	↑	↑	↑	↑	CD4+ IFNγ+ T cells ↓ Tregs
Fekete	C57/B6	PLX5622	M/F	16 d	*Herpesviridae*	PRV (i.p.)	-	↑	↑	↓	P2Y12 mediated phagocytosis
Seitz	SW	PLX5622	F	14 d	*Flaviviridae*	WNV (f.p.)	↑	↔	↑	↑	–
	SW	PLX5622	F	14 d	*Flaviviridae*	JEV (f.p.)	↑	↔	↑	↑	–
Waltl	C57/B6	PLX5622	F	21 d	*Picornoviridae*	TMEV (i.c.)	↑	↑	↑	↑	
Chhatbar	C57/B6	BLZ945	M/F	8 weeks	*Rhabdoviridae*	VSV (i.n.)	↑	n/r	↑	n/r	–
Funk	C57/B6	PLX5622	M	14 d	*Flaviviridae*	WNV (f.p.)	↑	↑	↑	n/r	↓CD8+ T cell activation
	C57/B6	PLX5622	M	14 d	*Flaviviridae*	WNV-E218A (i.c.)	↑	↑	↑	↓	
Sanchez	C57/B6	PLX5622	M	7 d	*Picornoviridae*	TMEV (i.c.)	↑	↑	↑^*^	n/r	–

*ICR, Institute of Cancer Research; CLD, clodronate liposomes; LCs, Langerhans cells in skin; SW, Swiss Webster; PVM, Perivascular macrophages; Mort., mortality; Morb., morbidity; WNV, West Nile virus; DENV, Dengue virus; MHV, Mouse hepatitis virus; PRV, pseudorabies virus; JEV, Japanese encephalitis virus; TMEV, Theiler's murine encephalomyelitis virus; CTL, Cytotoxic T lymphocyte; P2Y12, purinergic receptor P2Y12; i.c., intracranial; i.n., intranasal; i.p., intraperitoneal; f.p., footpad. n/a, not applicable; n/r, not reported*.

* *Viral antigen immunostaining*.

Importantly, microglial depletion was associated with a negative impact on clinically relevant endpoints such as neurological disease and/or death (58, 73–79)—even in the *Il34*^*LacZ*/*LacZ*^ model (68)—implying that microglia are critical to survival in encephalitis. In a study using a nonlethal pseudorabies virus (PRV) model (74), microglial depletion led to the development of overt neurological disease, which correlated with viral burden in the brain (74). Similarly, in other studies where neurological disease was reported, microglia-depleted mice showed increased disease severity prior to death (Table 1). Collectively, these studies establish clear links between viral resistance and negative disease outcomes, consistent with a protective function of microglia.

## Depletion of Microglia Impairs the Antiviral Function of Recruited T Cells

The mechanism(s) underlying these protective properties remain uncertain. Preliminary data suggest that the T cell response induced by microglia is involved, however the impact on the quality and magnitude of the antiviral T cell response was inconsistent across different models (76–78), and no impact on T cell immunophenotype was observed in others (74), meaning it is not yet possible to draw definitive conclusions.

Viral clearance and/or disease outcome relies on a functional T cell response in several mouse models of encephalitis (e.g., West Nile virus [WNV], mouse hepatitis virus [MHV], or Theiler's murine encephalomyelitis virus [TMEV]). In the TMEV model, strain-specific differences in disease phenotype are linked to the effectiveness of the CD8+ T cell response, which is subject to Treg suppression, and consequent clinical disease, in susceptible mouse genetic backgrounds (76). Although microglia depletion had no impact on CD8+ T cell recruitment, hippocampal Tregs were increased, and the disease phenotype recalled that seen in susceptible backgrounds (76). In contrast, in a WNV model where CD8+ T cells are also protective, an increased number of CD8+ T cells were recruited to the brain of microglia-depleted mice. However, these CD8+ T cells had a blunted activation phenotype, with reduced proportions of cells expressing the activation markers CD69 or CD160 (78). Whilst it is plausible that this would contribute toward a defect of viral control and/or clearance, due to the increase in overall CD8+ T cell recruitment in PLX5622-treated animals, the net effect was to achieve an equivalent number of activated CD8+ T cells in the brains of both groups (78).

In MHV, both CD8+ and CD4+ T cells are involved in viral clearance (80, 81), whilst CD4+ T cells also contribute to pathogenesis (82). In the MHV model, microglia depletion did not negatively impact CD8+ T cell recruitment, but was associated with significant reductions in the recruitment of CD4+ T cells and Tregs, and importantly in IFNγ expression by CD4+ T cells (77)—in complete contrast to TMEV (76). While the overall impact of these changes to both “effector” CD4+ T cell and Treg populations is difficult to reconcile, the authors concluded that the observed reduction in Treg recruitment might contribute to T cell immunopathology, thereby worsening disease outcome.

The collective implication of these studies is that microglia contribute positively to aspects of T cell recruitment and/or activation, however the precise nature of the protective T cell response induced by microglia requires further examination.

## CNS Intrinsic Protection by Microglia: Role of Phagocytosis

A mechanism by which microglia might mediate protection, independently of their effects on the T cell response, was reported in the PRV model. Here microglia were recruited toward and engulfed virus-infected neurones, a process that required microglial P2Y12 signaling (74). As stated above, the depletion of microglia led to the development of overt disease and increased viral replication. Similarly, in a study using an attenuated WNV strain, phagocytosis of presynaptic CA3 neurones in the hippocampus was observed, which depended on deposition of the complement protein C3 (69). *Il34*^*LacZ*/*LacZ*^ mice were included in this study to show that the attrition of presynaptic neurones was microglia-dependent. Since these findings were associated with defects of spatial orientation in recovered wild-type mice, the authors concluded that microglia contribute to pathogenesis. However, the outcome of infection in microglia-depleted *Il34*^*LacZ*/*LacZ*^ mice was not reported (69), whereas in a previous study using the same model there was significantly enhanced mortality in *Il34*^*LacZ*/*LacZ*^ (microglia-depleted) mice (68). Thus, it seems that while microglial phagocytosis may prevent mortality in the acute phase (68, 74), this might come at later stages at a cost of permanent neurological damage (69). Microglia respond to insults with a broad spectrum of activation states, and are implicated in not just the initiation, but also the resolution of inflammatory responses (55). As for phagocytosis, other aspects of the microglial response to infection might contribute to tissue damage in encephalitis models, as they do in models of sterile neuroinflammation (83, 84). Key questions, yet to be addressed in viral encephalitis models, are: (i) what is the effect of microglial depletion in the resolution phase, and (ii) does a “goldilocks zone” of microglial activity exist, in which *just enough* reaction is sufficient for protection without incurring permanent neurological damage, but where both *too little* and *too much* might contribute adversely to encephalitis outcome. In this scenario, therapeutic approaches to either boost or suppress microglial responses, at different stages of disease, might prove beneficial.

## Enhanced or Reduced Inflammation in Microglia Depleted Brains?

As discussed above, clinical studies have reported associations between inflammatory cytokine production and adverse outcomes. Interestingly, despite the suspicion that microglia are central to pathogenic neuroinflammation, microglial depletion was unexpectedly associated in some studies with an increase in the synthesis of cytokines and/or chemokines in the brain of infected animals (75–77), whereas in other studies there was a reduction (73, 74, 78). There is no definitive explanation for these contradictory observations. One possibility is the increased recruitment of circulating monocytes to microglia-depleted brains in circumstances where cytokine/chemokine synthesis was enhanced (76, 77), which was not observed in other studies (58, 74, 78)—including ones in which a reduction in cytokine/chemokine induction was reported (78). However, these factors were not consistently reported, and furthermore, whether this apparent association is causal remains uncertain. Other possible explanations include:

- a correlation between increased viral replication and inflammation–noted in one of these studies (77) and previously reported elsewhere (85, 86);
- the activation of CNS-resident cell types, such as astrocytes, which was similarly noted in one study (76), as in other disease models (87, 88).
- the loss of an immunoregulatory function of microglia, as mentioned above.

Overall, further work is needed to clarify the potential relationship between microglial depletion and immune dysregulation in the CNS.

## Non-specific Effects of CSF1R Inhibition: a Confounding Factor?

There are also outstanding questions regarding the specificity of CSF1R blockade for microglia. Whilst PLX5622 (71) and BLZ945 (89) are more specific for CSF1R than earlier inhibitors (63), PLX5622 has been shown to deplete macrophages from various tissues, including kidney (90) and peripheral nervous system (91), leaving open the possibility of non-specific depletion of macrophages either in the CNS (e.g., meningeal or perivascular macrophages) or elsewhere. In encephalitis models, depletion of CNS macrophages (CD45^hi^ CD11b^+^) was reported in some instances (58, 78), although not in several others (74, 76, 77, 79). Again, this inconsistency is unexplained, but one possibility is the use of CD45/CD11b expression to distinguish CNS macrophages from microglia (84, 92). Increased microglial CD45 expression has been noted during VSV encephalitis (58), which might confound assessment of the effect of PLX5622 on CNS macrophages. Importantly, any depletion observed was minor compared to the effects on microglia (58, 78). The use of more specific markers of microglia (e.g., TMEM119) or perivascular macrophages (e.g., CD206) might help to resolve this issue (93, 94).

In the studies which quantified effects on systemic myeloid populations (58, 76–78) there were no reductions in splenic or bone marrow macrophages (74, 76–78), however variable effects on circulating monocytes were reported, with reductions in some studies (76, 78), but no effect in others (58, 74, 77). Any impact on systemic myeloid populations is especially problematic for flavivirus models, where systemic viral replication occurs as a precursor to CNS neuroinvasion (75, 78); systemic depletion of myeloid cells might (i) enhance systemic viral replication, in turn enhancing the load of virus reaching the CNS, and/or (ii) cause CNS-extrinsic disease that might contribute independently to mortality. Funk et al. reported an enhancement of systemic WNV replication and clinical disease in association with PLX5622-induced monocyte depletion (78). To overcome this, they also challenged mice via the intracranial route, confirming a negative impact of microglial depletion (78), consistent with previous findings (68). By contrast, Seitz et al., in the same WNV model, did not detect systemic viral replication (75). The extent to which possible “off-target” effects of CSF1R inhibition might confound data generated in encephalitis models remains uncertain.

## Conclusion

Through the recent use of microglial depletion systems, it is evident that microglia play an essential protective function in mouse models of viral encephalitis. Nevertheless, important questions remain about the mechanism(s) by which microglia (and possibly other CNS resident macrophages) mediate these protective effects (Figure 1). An intriguing observation–that microglial depletion leads in some circumstances to enhanced neuroinflammation, hints at a possible regulatory function of microglia in encephalitis and is an important area for future investigation. Another priority area is the possibility that dysregulated microglial responses, whilst protective against mortality in the early stages of encephalitis, might contribute at later stages to permanent neurological sequelae. A key question is whether these various findings in mouse models translate to human systems; the recent development of methods of microglial differentiation from human pluripotent stem cells offers a potential solution to this question [reviewed in (46)]. Collectively, these observations suggest the exciting possibility that (i) defects in the microglial response might underlie encephalitis susceptibility in patients, and (ii) that targeting this response may provide new therapeutic opportunities.

## Author Contributions

CD: conceptualization and funding acquisition. CH and CD: literature searching, manuscript writing, and figures and tables.

### Conflict of Interest

The authors declare that the research was conducted in the absence of any commercial or financial relationships that could be construed as a potential conflict of interest.
